# Treatment of pure red cell aplasia in a chronic kidney disease patient with roxadustat: A case report

**DOI:** 10.1515/biol-2025-1155

**Published:** 2025-10-04

**Authors:** Shanlin Liu, Bonan Yan, Yuhua He

**Affiliations:** Department of Nephrology, Hospital of Chengdu University of Traditional Chinese Medicine, No. 39 Shi-er-Qiao Road, Chengdu, 610072, Sichuan, China; Department of Nephrology, Nanbu County Hospital of Traditional Chinese Medicine, Nanchong, 637300, Sichuan, China

**Keywords:** roxadustat, pure red cell aplasia, chronic kidney disease, renal anemia

## Abstract

Pure red cell aplasia (PRCA) is a rare blood disorder that is characterized by severe hypo-erythroid bone marrow hypoplasia leading to severe anemia that usually does not respond to standard treatment. In patients with chronic kidney disease (CKD), especially in those patients who are erythropoiesis-stimulating agent (ESA) resistant, management can be quite difficult. In this case report, we will present a 67-year-old woman with CKD (not on dialysis) who presented with refractory anemia (hemoglobin 45–65 g/L) and was ultimately diagnosed with PRCA after bone marrow aspirate. This patient had previously received treatment (ESAs, iron supplementation, and multiple blood transfusions) without improvement in her blood counts. After initiation of oral Roxadustat 100 mg t.i.w., her hemoglobin increased gradually and stabilized between 100 and 106 g/L, and she no longer required blood transfusions. This case report highlights the potential role of Roxadustat as a new therapeutic option for PRCA and ESA-resistant CKD. While these data are encouraging, larger controlled studies are going to be required to evaluate measures of efficacy, dosing, and long-term safety in this population.

## 1 Introduction

Pure red cell aplasia (PRCA) is a rare hematological condition that presents as severe reticulocytopenia and marked reduction of erythroblasts in the bone marrow [1]. PRCA can be congenital or acquired, and the acquired type of PRCA is sometimes associated with autoimmune disease, viral infections, malignancies, and certain medications [2]. In patients suffering from chronic kidney disease (CKD), PRCA can complicate the already impaired erythropoiesis from decreased erythropoietin (EPO) secretion and production [3]. The cornerstone of treatment for PRCA includes glucocorticoids, immunosuppressive medications, or erythropoiesis-stimulating agents (ESAs), although many patients have little or no response [4,5]. For cases of PRCA that are refractory, there could be the option of splenectomy, thymectomy, and hematopoietic stem cell transplantation, but these could pose a significant risk [6]. Blood transfusions provide symptomatic relief but may carry the risks of iron overload [7].

Genetic influences may also be associated with the development and progression of CKD and anemia, especially in populations with predisposed variations of iron regulation/erythropoiesis-related genes [8].

We chose this case due to clinical rarity, extremely refractory to recommended treatment, and notable hematologic improvement experienced with Roxadustat, thus providing evidence for a new potential treatment. Roxadustat is a hypoxia-inducible factor prolyl hydroxylase inhibitor (HIF-PHI), which stimulates endogenous EPO production, regulates iron metabolism, and promotes erythropoiesis [9]. Roxadustat stabilizes hypoxia-inducible factors (HIFs) as a contrast to ESAs, which stimulate erythropoiesis even in the context of inflammation or ESA resistance [10]. There are limited clinical data on the use of Roxadustat in PRCA, particularly in adult patients with CKD not on dialysis, and this case can provide useful insights into the potential use of Roxadustat.

## 2 Case presentation

### 2.1 Patient background and initial presentation

A 67-year-old female patient with a background of hypertension was brought to a county-level hospital in June 2020 due to complaints of fatigue and chest tightness. At admission, laboratory studies indicated severe anemia, with hemoglobin levels of 69 g/L, RBC count of 2.17 × 10¹²/L, and a hematocrit level of 22%. The MCV was recorded at 101.1 fL and MCHC 312 g/L. Platelet counts were moderately low at 118 × 10⁹/L. Other biochemistry investigations showed high transferrin saturation (95.15%) and elevated ferritin (741.40 ng/mL), suggesting possible iron overload. Additionally, kidney panel tests indicated increased serum creatinine (326 μmol/L) and urea concentrations (18.45 mmol/L), consistent with CKD.

After the first diagnosis, the patient was subsequently prescribed Ferrous Succinate Sustained Release Tablets (0.2 g per day) and Yiqi Weixue Capsules (1.35 g, three times a day). Yiqi Weixue capsules are an example of traditional Chinese medicine, which contains *Astragalus membranaceus* and *Angelica sinensis* to help enhance hematopoiesis and promote blood circulation. After 1 month of treatment, there was no change in hemoglobin levels, so another line of therapy was warranted.


**Informed consent:** Informed consent has been obtained from all individuals included in this study.
**Ethical approval:** The research related to human use has been complied with all the relevant national regulations and institutional policies and in accordance with the tenets of the Helsinki Declaration and has been approved by the Ethics Committee of the Hospital of Chengdu University of Traditional Chinese Medicine.

### 2.2 Progression and initial treatment attempts

The patient was started on recombinant human EPO (EPIAO, 20,000 IU, twice a week) with monthly transfusions of euphoric red blood cell suspension (1.0–2.0 units for each transfusion) for the next ten months. Unfortunately, despite these interventions, her hemoglobin levels continued to swing up and down, ranging from 44–60 g/L. This ongoing anemia that was resistant to ESA therapy and transfusion led to concerns for possible erythropoietic failure, possibly from a marrow agent. In March 2021, Roxadustat was introduced at a low dose of 100 mg once a week as another option. One month post-initiation, there was no discernible hematology response, and she was sent to the Hematology Department of our hospital in May 2021 for further treatment (Table 1).

**Table 1:** Key laboratory trends over time

Date	Hemoglobin (g/L)	Ferritin (ng/mL)	Treatment changes
March 2021	55	1,925	ESA + transfusions
Nov 2022	77	855.1	Roxadustat (100 mg three times/week)
Aug 2023	100–106	513.3	Stable outcome

### 2.3 Hematologic evaluation and PRCA diagnosis

Based on the clinical and lab work, the patient was seen on the other side of the track. On physical examination, she was quite pale, without any signs of mucosal or cutaneous bleeding. No lymphadenopathy could be palpated; cardiovascular examination showed a heart rate of 87 beats per minute with a regular rhythm. There was no evidence from the examination of her legs suggesting volume overload contributed to her anemia. At the time of referral, she had worsening anemia with decreased hemoglobin at 55 g/L and reticulocyte % of 0.11%. The reticulocyte count was extremely low, at 2 × 10⁹/L, confirming very severe suppression of erythropoiesis. Serum ferritin increased to 1,925 ng/mL, further substantiating the iron overload. Renal function tests were still abnormal: creatinine at 305.3 μmol/L and cystatin C at 3.69 mg/L.

The patient had a bone marrow aspiration and biopsy to evaluate the cause of the refractory anemia. Morphological examination showed erythroid hypoplasia, with the granulocyte precursors making up 84% of the nucleated cells and only minimal amounts of the erythroid precursors. The myeloblasts were 0.5%, and the megakaryocytes were normal in morphology. The biopsy showed PRCA. Flow cytometric analysis and other work-ups were negative for neoplastic, autoimmune, and parvovirus B19 causes.

### 2.4 Treatment modifications and response to therapy

After the diagnosis of PRCA with underlying CKD, the treatment plan was revised. Cyclosporine was no longer an option, given the patient’s renal impairment, and initiation of Prednisone (5 mg/day) and a number of other medications were started, i.e., α-Ketoacid Tablets, Med Charcoal Tablets, Nifedipine Controlled Release Tablets, Lansoprazole Enteric Coated Tablets, and Deferasirox Dispersible Tablets. Even with the medications changed around, hemoglobin levels remained very low (45–65 g/L), requiring ongoing transfusions.

In November 2022, given the ongoing anemia and my lack of sustained response, Roxadustat was reintroduced at a higher professed dose of 100 mg t.i.w. Hemoglobin began to improve slowly over the next few months, reaching a level of 100 and then stabilizing at 106 g/L. In tandem, serum ferritin levels began to decline from 855.1 to 513.3 ng/mL, suggesting an improvement in iron metabolism. During this entire timeframe, the patient was free from transfusions, thromboembolic complications, or any significant reno- or hepato-toxicity.

### 2.5 Key laboratory trends over time

**Figure 1:**
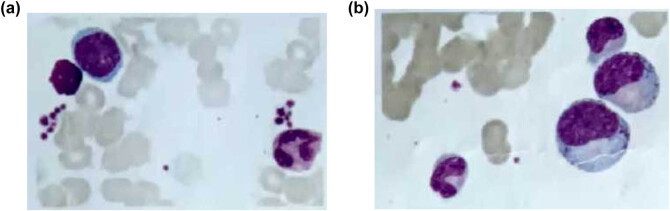
Morphological analysis of bone marrow cells. (a and b) Bone marrow smears showing reduced erythroid precursors, with normal granulocyte and megakaryocyte morphology, support the diagnosis of pure red cell aplasia (PRCA).

**Figure 2:**
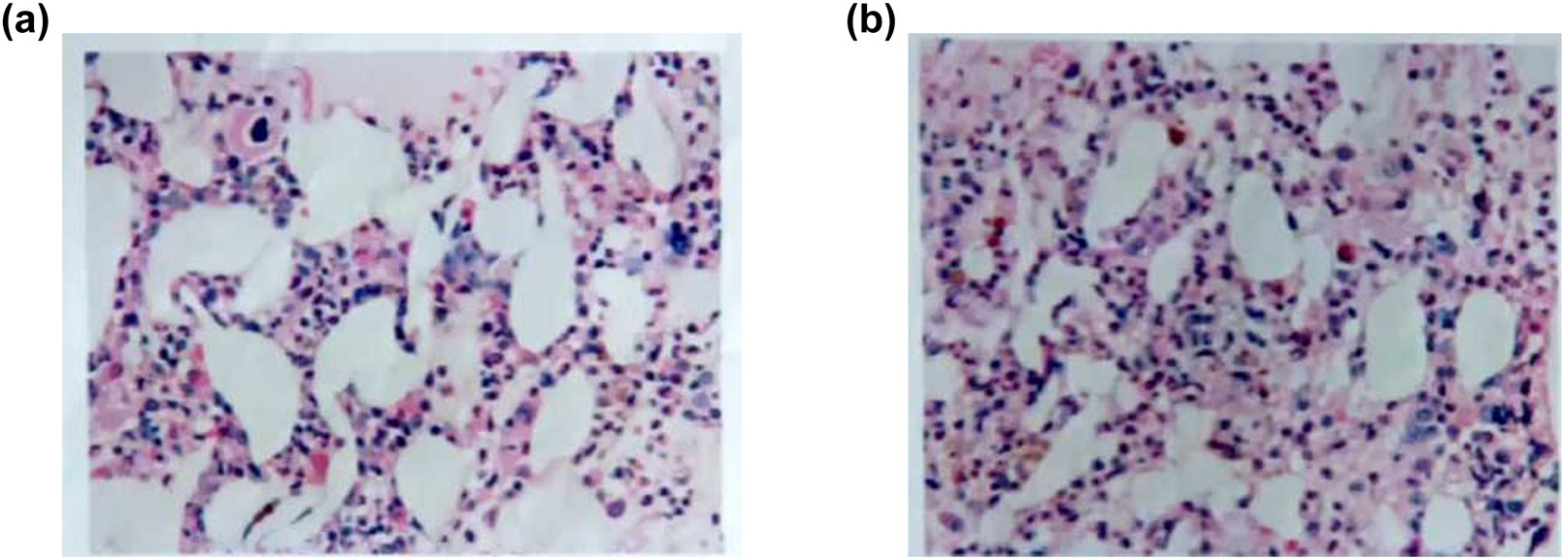
Pathological diagnosis of aspirated bone marrow tissue. (a and b) Bone marrow biopsy revealing low hematopoietic tissue hyperplasia, marked erythroid hypoplasia, and preserved myeloid and megakaryocyte lineages, consistent with PRCA.

**Figure 3:**
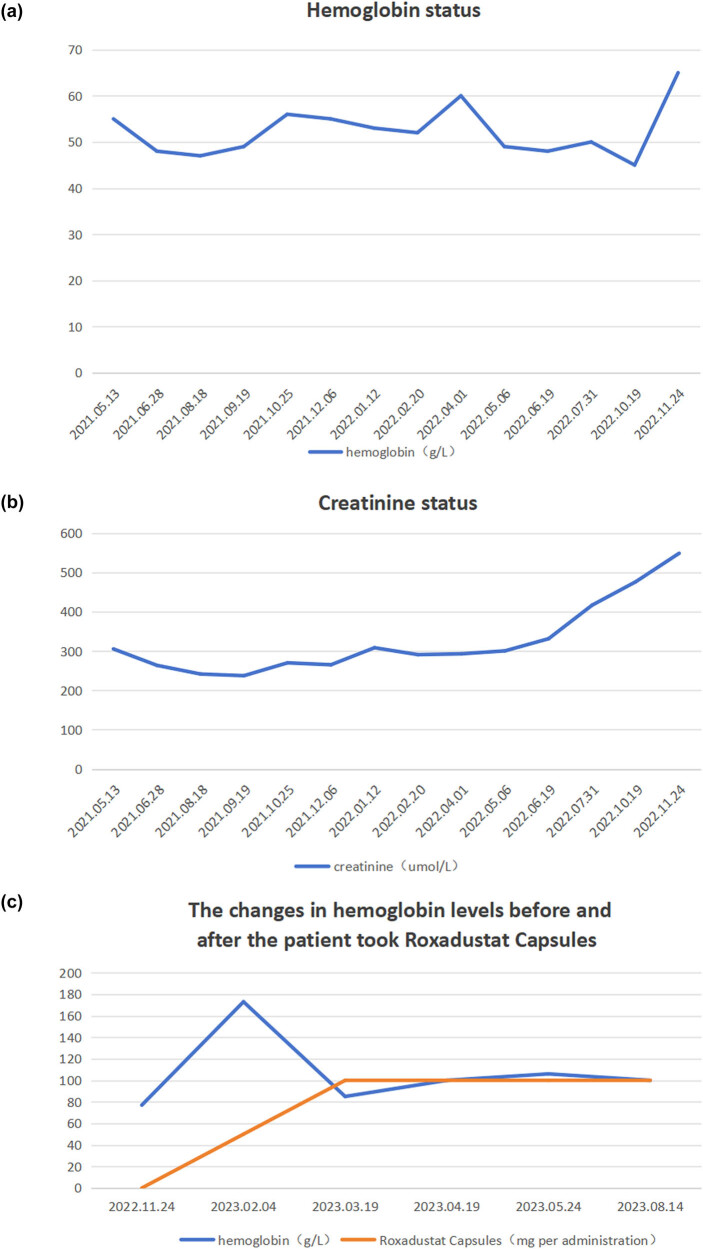
Laboratory parameter trends. (a) Hemoglobin status over time, showing initial low levels and subsequent improvement following Roxadustat therapy. (b) Creatinine levels reflecting renal function status throughout treatment. (c) Hemoglobin changes before and after Roxadustat administration, illustrating treatment response and stabilization of anemia.

The results indicate that a greater and sustained dose of Roxadustat was necessary for hematologic recovery. Compared to the previous trial (March 2021) study (hemoglobin 55 g/L, ferritin 1,925 ng/mL), the modified regimen not only improved hemoglobin (77 g/L by November 2022) but also stabilized hemoglobin (100–106 g/L by August 2023) with trending decrease in ferritin (855.1–513.3 ng/mL) indicating that the patient was becoming less erythroid suppressive with improved erythropoiesis (making use of iron). Figure 1a and b shows a bone marrow smear and erythroid suppression, Figure 2a–b shows biopsy-confirmed PRCA, and Figure 3a–c shows the hematologic function and renal function trends post-Roxadustat treatment.

## 3 Results

### 3.1 Hematologic response to roxadustat treatment

After starting treatment with high-dose Roxadustat at 100 mg administered three times per week, the patient’s hemoglobin concentration increased steadily. Patient’s pre-treatment hemoglobin was 77 g/L as of November 2022. Following continuous dosing until March 2023, hemoglobin concentration was 85 g/L. Hemoglobin concentration remained stable by April 2023 in the range of 100–106 g/L and continued to remain essentially the same through August 2023 (Figure 3c). During this time, serum ferritin concentration dropped from a high of 855.1–513.3 ng/mL with meaning the patient was able to improve its iron utilization and erythropoiesis (Table 1). Most importantly, the patient no longer required red blood cell transfusions, showing that Roxadustat was effective for the patient in obtaining transfusion independence.

### 3.2 Comparison of initial and adjusted roxadustat therapy

The initial trial of Roxadustat in March 2021, at 100 mg once a week, found no hematologic benefit; hemoglobin was still at 55 g/L, with ferritin rising to 1.925 ng/mL, indicating ineffective erythropoiesis. This could be indicative of underdosing or an underlying pathologic disturbance, such as PRCA was not addressed. PRCA was subsequently confirmed, and the dose was escalated in November 2022 (100 mg three times weekly). There was a dramatic and sustained increase in hemoglobin concentration consistent with a dose response in cases of PRCA, also with CKD. This program also allowed the patient to discontinue transfusions.

### 3.3 Morphological analysis of bone marrow cells

Bone marrow aspiration showed severe erythroid hypoplasia with few erythroid precursors, and 84% of nucleated cells constituted granulocyte precursors (Figure 1a and b). Myeloblasts were noted at 0.5%, and the megakaryocytes were morphologically normal. There was no fibrosis or dysplastic changes, meaning other marrow pathology was effectively ruled out.

### 3.4 Histopathological evaluation of bone marrow biopsy

A bone marrow biopsy showed severely decreased hematopoiesis with only 35% hematopoietic cellularity and 65% adipocytes. The granulocyte-to-erythrocyte ratio was very high due to severe depletion of all erythroid elements and was consistent with PRCA. After visualizing the smear, there were occasions of clusters of granulocytes, while cells from the erythroid lineage were rarely noted. Megakaryocytes were noted at a frequency of 3–6 per high-power field and appeared normative. There were no signs of myelofibrosis or malignancy, or infiltrative processes (Figure 2a and b).

### 3.5 Renal function and safety outcomes

During high-dose Roxadustat treatment, the patient’s renal function remained stable without evidence of acute kidney injury. Serum creatinine (Figure 3b) and no hepatic toxicity, thromboembolic complications or other side effects were documented. This is supportive of Roxadustat’s tolerability and safety in this CKD-PRCA case.

## 4 Discussion

PRCA is a rare hematological disease characterized by selective erythroid aplasia and reticulocytopenia, often resulting from autoimmunity, virus infections, malignancies, or medication exposure [11,12]. In patients with CKD, the situation is complicated by low endogenous EPO production, causing a more challenging anemia to treat [13]. The case described below highlights the significance of this dual treatment burden experienced by a CKD patient, who remained transfusion dependent despite being on recombinant human EPO and iron supplementation, and subsequently diagnosed with PRCA by bone marrow biopsy.

Standard immunosuppressive treatment (IST) for patients with PRCA, including corticosteroids, cyclosporine A, and sirolimus, is a widely used therapy; however, as outlined above, IST has a high side effect profile and cannot be used in some patients due to nephrotoxicity and recurring complications from immunosuppression [5,14,15]. In our case, IST was not an option due to renal dysfunction, so we had to follow a different approach. Roxadustat, a HIF-PHI, is an exciting contemporary option to treat renal anemia because it is a non-immunosuppressive therapeutic option whereby Roxadustat, through the stabilization of HIF-α, stimulates endogenous EPO production and iron metabolism while inhibiting hepcidin [16,17,18,19,20,21,22].

Furthermore, Roxadustat may be able to circumvent the immune-mediated suppression of erythropoiesis, which makes it attractive for patients with EPO-resistant PRCA. Recent evidence suggests that Roxadustat has potential benefits in this regard. Zheng et al. [23] and Fu et al. [24] demonstrated resolution of anemia in patients with CKD and PRCA who had no EPO response. Wan et al. [25] reported a patient with anti-EPO antibody-mediated PRCA who had improved clinical outcomes using Roxadustat. In our case report, we also observed an initial rise in hemoglobin from 77 to 100–106 g/L following initiation of high-dose Roxadustat, with transfusion independence.

Zhao et al. showed that Roxadustat promotes erythropoiesis in ESA-resistant patients by altering pro-inflammatory cytokines and downregulating the hepcidin expression, suggesting a role in immune-refractory anemia [26]. Liu et al. determined that the dose-dependence of Roxadustat was also strongly associated with faster erythroid recovery, in dialysis and non-dialysis patients [27]. These studies further strengthen the notion that Roxadustat can have utility beyond the classical mechanisms of action offered by ESA therapy.

On the flipside, we need to consider the limitations of the approach. First, this is a single-patient case report, which limits generalizability. Second, there was no comparator group, which prevents concluding efficacy. Third, we do not clearly understand the long-term safety of Roxadustat to treat PRCA, especially concerning thromboembolic events.

Future research should focus on multicenter and randomized controlled trials of Roxadustat and IST in the clinical management of patients with PRCA, particularly among those with concurrent CKD. Future molecular studies may help to identify additional pathways that Roxadustat affects beyond HIF stabilization and provide more comprehensive avenues for patient selection and dosing strategies.

## 5 Conclusions

This case emphasizes the promise of Roxadustat as a therapeutic option for PRCA in a CKD patient who had failed all other conventional therapies. A higher dosing strategy with Roxadustat (100 mg three times weekly) provided hemoglobin stabilization, and transfusions were eliminated. While this outcome is promising, this is a single case, which cannot define general efficacy, so larger and controlled studies are required to characterize a therapeutic effect with Roxadustat for PRCA. If a timely diagnosis is made, consideration of alternative therapies such as Roxadustat could provide a clinical advantage in similarly refractory cases.

## References

[1] Means Jr RT. Pure red cell aplasia. Hematol Am Soc Hematol Educ Program. 2016;2016(1):51–6. 10.1182/asheducation-2016.1.51. PMC6142432 27913462

[2] Kaito K, Otsubo H, Usui N, Kobayashi M. Diagnosis and treatment of pure red-cell aplasia. Blood Transfus. 2022;20(3):207–14. 10.2450/2022.0173-21.

[3] Erslev AJ. Erythropoietin and the regulation of erythropoiesis. N Engl J Med. 1991;324(19):1339–44. 10.1056/NEJM199105093241906. 2017231

[4] Brodsky RA. How I treat acquired pure red cell aplasia. Blood. 2021;137(15):1991–8. 10.1182/blood.2020008394. PMC8057257 33657207

[5] Sawada K, Fujishima N, Hirokawa M. Acquired pure red cell aplasia: updated review of treatment. Br J Haematol. 2008;142(4):505–14. 10.1111/j.1365-2141.2008.07217.x. PMC2592349 18510682

[6] Maekawa T, Tanaka T, Kameoka J. Splenectomy as a treatment for pure red cell aplasia: a case series and literature review. Int J Hematol. 2020;111(1):93–101. 10.1007/s12185-019-02764-8.

[7] Cazzola M, Malcovati L. Myelodysplastic syndromes—coping with ineffective hematopoiesis. N Engl J Med. 2005;352(6):536–8. 10.1056/NEJMcibr045502. 15703418

[8] Kumari R, Tiwari S, Atlani M, Anirudhan A, Goel SK, Kumar A. Association of single nucleotide polymorphisms in KCNA10 and SLC13A3 genes with the susceptibility to chronic kidney disease of unknown etiology in central indian patients. Biochem Genet. 2023;61(4):1548–66. 10.1007/s10528-023-10335-7. 36696070

[9] Locatelli F, Fishbane S, Block GA, Macdougall IC. Targeting hypoxia-inducible factors for the treatment of anemia in chronic kidney disease patients. Am J Nephrol. 2017;45(3):187–99. 10.1159/000455382. 28118622

[10] Chaparro JA, Kazmi I, Hesson A, Gupta V. Roxadustat: an emerging treatment for anemia associated with chronic kidney disease. J Clin Pharm Ther. 2021;46(4):898–906. 10.1111/jcpt.13360.

[11] Lobbes H. L’érythroblastopénie: diagnostic, classification, traitement [pure red cell aplasia: diagnosis, classification and treatment]. Rev Med Internet. 2023 Jan;44(1):19–26. 10.1016/j.revmed.2022.10.385. Epub 2022 Nov 3. PMID: 36336519. 36336519

[12] Heras-Benito M. Renal anemia: current treatments and emerging molecules. Rev Clin Esp. 2023 Aug–Sep;223(7):433–9. 10.1016/j.rceng.2.023.06.006. Epub 2023 Jun 20. PMID: 37348652. 37348652

[13] Alfaraj M, Saeed HA. Pure red cell aplasia is a disease of great diversity. J Appl Hematol. 2020;11(1):1. 10.4103/joah.joah_63_19.

[14] Red Blood Cell Disease (Anemia) Group, Chinese Society of Hematology, Chinese Medical Association. [Chinese expert consensus on the diagnosis and treatment of acquired pure red cell aplasia (2020)]. Zhonghua Xue Ye Xue Za Zhi. 2020 Mar;41(3):177–84. doi: 10.3760/cma.j.issn.02532727.2020.03.001. 10.3760/cma.j.issn.0253-2727.2020.03.001 PMC7357928 32311886

[15] Yali D. In vitro exploratory study on the clinical efficacy of sirolimus in the treatment of refractory pure red cell aplastic anemia and its mechanism of action [D]. Beijing, China: Peking Union Medical College; 2017.

[16] Red cell diseases (anemia) Group, Society of Hematology. Chinese Medical Association. Chinese expert consensus on the diagnosis and treatment of acquired pure red cell aplasia. Chin J Hematol. 2020;41(3):177–84. 10.3760/cma.j.issn.0253.2727.2020.03.001. PMC7357928 32311886

[17] Chen Z, Liu X, Chen M, Yang C, Han B. Successful sirolimus treatment of patients with pure red cell aplasia complicated with renal insufficiency. Ann Hematol. 2020 Apr;99(4):737–41. 10.1007/s00277-020-03946-2 32030447

[18] Brown TJ, Mamtani R, Bange EM. Immunotherapy adverse effects. JAMA Oncol. 2021 Dec;7(12):1908. 10.1001/jamaoncol.2021.5009. 34709372

[19] Swinson B, Waters PS, Webber L, Nathanson L, Cavallucci DJ, O’Rourke N, et al. Portal vein thrombosis following elective laparoscopic splenectomy:incidence and analysis of risk factors. Surg Endosc. 2022 May;36(5):3332–9. 10.1007/s00464-021-08649-x. 34331132

[20] Xavier RD, Devaraj S, Sadasivam V, Prakasam O, Menon N, Hariharan A, et al. Thymoma associated with pure red cell aplasia: a case report and literature review.Indian. J Thorac Cardiovasc Surg. 2020 Jul;36(4):404–8. 10.1007/s12055-019-00875-2.Epub. PMC7525650 33061149

[21] Taher AT, Weatherall DJ, Cappellini MD. Thalassaemia. Lancet. 2018 Jan;391(10116):155–67. 10.1016/S0140-6736(17)31822-6. 28774421

[22] Parisi S, Finelli C, Fazio A, De Stefano A, Mongiorgi S, Ratti S, et al. Clinical and molecular insights in erythropoiesis regulation of signal transduction pathways in myelodysplastic syndromes and β-thalassemia. Int J Mol Sci. 2021 Jan;22(2):827. 10.3390/ijms22020827. PMC7830211 33467674

[23] Zheng G, Chang X, Gao W, Xue H. A case of uremia combined with pure erythrocyte aplastic anemia treated with roxarestat combined with immunosuppressant. Chin J Nephrol. 2021;37(1):57–8. 10.3760/cma.j.cn441217-20200429-00011.

[24] Fu J, Qin Z, Yang W, Meng L, Wang S. Roxarestat for the treatment of a case of aplastic anaemia in dialysis patients with chronic kidney disease combined with pure red blood cells. J Clin Nephrol. 2023;23(7):613–6. 10.3969/j.issn.1671-2390.2023.07.015.

[25] Wan K, Yin Y, Luo Z, Cheng J. The remarkable response to roxadustat in a case of anti-erythropoietin antibody-mediated pure red cell aplasia. Ann Hematol. 2021;100(2):591–3. 10.1007/s00277-020-04269-y. 32940725

[26] Zhao XN, Liu SX, Wang ZZ, Zhang S, You LL. Roxadustat alleviates the inflammatory status in patients receiving maintenance hemodialysis with erythropoiesis-stimulating agent resistance by increasing the short-chain fatty acids producing gut bacteria. Eur J Med Res. 2023;28(1):230. 10.1186/s40001-023-01179-3. PMC10331997 37430374

[27] Liu J, Zhou K, Meng C, Liu Z, Huang R, Waheed Y, et al. Roxadustat effectiveness versus ESAs in peritoneal dialysis patients during the COVID-19 pandemic: A retrospective study. PLoS One. 2025;20(3):e0320536. 10.1371/journal.pone.0320536. PMC11940824 40138338

